# Endolymphatic Hydrop Phenotype in Familial Norrie Disease Caused by Large Fragment Deletion of *NDP*

**DOI:** 10.3389/fnagi.2022.771328

**Published:** 2022-04-18

**Authors:** Yuerong Gong, Zhang Liu, Xiaolin Zhang, Shuang Shen, Qijun Xu, Hongchun Zhao, Jing Shang, Weiguo Li, Yanfei Wang, Jun Chen, Xiuzhen Liu, Qing Yin Zheng

**Affiliations:** ^1^Department of Ophthalmology, Binzhou Medical University Hospital, Binzhou, China; ^2^Department of Otolaryngology Head and Neck Surgery, Institute of Otolaryngology, Binzhou Medical University Hospital, Binzhou, China; ^3^Institute of Hearing and Speech Rehabilitation, College of Special Education, Binzhou Medical University, Yantai, China; ^4^Medical Research Center, Binzhou Medical University Hospital, Binzhou, China; ^5^Department of Otolaryngology Head and Neck Surgery, Case Western Reserve University, Cleveland, OH, United States

**Keywords:** norrie disease, *NDP*, gene deletion, hearing loss, endolymphatic hydrops

## Abstract

Norrie disease (ND; OMIM 310600), a rare X-linked recessive genetic disorder, is characterized by congenital blindness and occasionally, sensorineural hearing loss, and developmental delay. The congenital blindness of ND patients is almost untreatable; thus, hearing is particularly important for them. However, the mechanism of hearing loss of ND patients is unclear, and no good treatment is available except wearing hearing-aid. Therefore, revealing the mechanism of hearing loss in ND patients and exploring effective treatment methods are greatly important. In addition, as a serious monogenic genetic disease, convenient gene identification method is important for ND patients and their family members, as well as prenatal diagnosis and preimplantation genetic diagnosis to block intergenerational transmission of pathogenic genes. In this study, a Norrie family with two male patients was reported. This pedigree was ND caused by large fragment deletion of *NDP* (norrin cystine knot growth factor NDP) gene. In addition to typical severe ophthalmologic and audiologic defects, the patients showed new pathological features of endolymphatic hydrops (EH), and they also showed acoustic nerves abnormal as described in a very recent report. PCR methods were developed to analyze and diagnose the variation of the family members. This study expands the understanding of the clinical manifestation and pathogenesis of ND and provides a new idea for the treatment of patients in this family and a convenient method for the genetic screen for this ND family.

## Introduction

Norrie disease (ND) is a rare X-chromosome-linked recessive genetic disease, mainly characterized by congenital blindness. Almost all patients are male, and the incidence rate is approximately 1/100,000 (Rodriguez-Munoz et al., 2018). ND patients develop retroretinal masses called pseudogliomas, which leads to blindness, due to the degenerative and proliferative changes in the retina. In addition, approximately 50% of patients show progressive neurological diseases, such as cognitive impairment and behavioral abnormalities. Approximately, one third of patients develops sensorineural hearing loss in their 20 s (Warburg, 1975). Some patients have more complex symptoms, including microphthalmia, developmental delay, and epilepsy. Patients also have other eye problems in infancy, such as corneal leukoplakia, iris atrophy, retroretinal fibrosis, vitreous hemorrhage, and retinal detachment (Lev et al., 2007; Okumura et al., 2015). Peripheral vascular disease consisting of leg ulcers and varicose veins has been described in a small number of patients (Michaelides et al., 2004; Smith et al., 2012).

The *NDP* (norrin cystine knot growth factor NDP) gene mutation is the only genetic factor of ND (Talebi et al., 2018). *NDP* maps to chromosome Xp11.2–11.3 and has three exons with a 1.85 kb transcript. The protein Norrin encoded by *NDP*, containing 133 amino acids, is expressed in the retina, choroid, and brain (Sims et al., 1992). Norrin is a secretory protein rich in cysteine junction motif (Andarva et al., 2018) and is mainly secreted by Muller cells and partly by retinal epithelial cells; Norrin is also found in retinal macrophages (Seitz et al., 2010; Chen et al., 2011). Norrin, as the ligand of the Wnt pathway receptor FZD4, can regulate the angiogenesis of related organs by activating the Wnt signaling pathway. Mutation or deletion of this protein can lead to eye diseases, such as ND (Xu et al., 2004; Chang et al., 2015).

The congenital blindness of ND patients is almost untreatable; thus, hearing is particularly important for them. However, the mechanism of hearing loss of ND patients is unclear, and no good treatment is available in addition to hearing-aid. Therefore, revealing the mechanism of hearing loss in ND patients and seeking treatment methods are greatly important. In addition, as a serious monogenic genetic disease, convenient gene identification method is greatly important for ND patients and their family, as well as prenatal diagnosis and preimplantation genetic diagnosis to block intergenerational transmission of the pathogenic genes. In this study, a large fragment deletion in *NDP* was identified in a Han family with ND. In addition to the reported ophthalmologic and audiology defects, the patients of this family showed new characteristics of hearing loss and cochlear damage, and they also showed acoustic nerves abnormal. At the same time, we developed PCR method to diagnose the variation of the family members. This study expands the understanding of the clinical manifestations and pathogenesis of ND, and provides a new idea for the treatment of the patients and a convenient method for the diagnosis of this family and other hereditary diseases with large fragment gene deletion in X-chromosome.

## Materials and Methods

### Family Introduction and Clinical Examination

**FIGURE 1:**
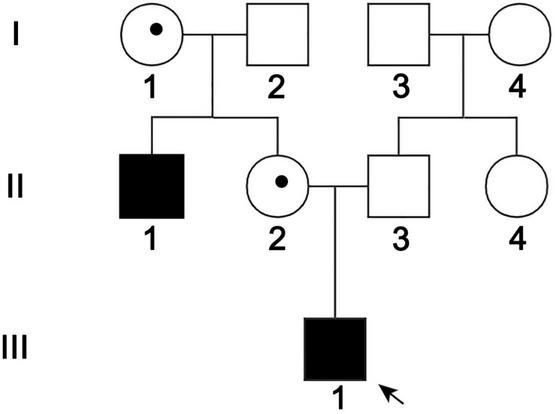
Pedigree of the ND family. Black arrow: the propositus. II-1 and III-1 were patients, I-1 and II-2 were female carriers, and the others were normal members.

The propositus and his family came from Shandong Province, China. They signed an informed consent form. The pedigree was shown in Figure 1. The study was approved by the Ethics Committee of the Binzhou Medical University Hospital (LW-020). On the principle of necessity and fitness, a full ophthalmic and audiology examination was performed. Eye B-ultrasound, anterior segment photography and Cranial CT inspections of the ears and eyes for individuals of II-1, II-2, and III-1; ear pure tone audiometry, auditory brainstem response (ABR) examination for III-1, electrocochleography (ECochG), distortion product otoacoustic emission (DPOAE) examination for II-1 and III-1, multiple stimuli auditory steady-state response (m-ASSR) examination for II-1. Cognitive functions were assessed through Mini-mental state examination (MMSE), Montreal cognitive assessment (MoCA) scales for III-1, and craniocerebral magnetic resonance imaging (MRI) for II-1, internal auditory canal MRI and Gadolinium-enhanced MRI of temporal bone for III-1. At the same time, peripheral vascular, growth, and development inspections were performed for I-1, I-2, II-1, II-2, II-3, and III-1, and the venous whole blood samples were collected for genetic testing from these individuals of the family.

### Whole Exome Sequencing and Bioinformatics Analysis

Genomic DNA was extracted from the peripheral blood by a blood genome extraction kit (TIANGEN, DP318). WES was performed at the Beijing Mykino Medical Laboratory (Beijing, China), and the exons were captured using Agilent SureSelect Human All Exon V6 kit, and high-throughput sequencing was performed using Illumina HiSeq X-10 sequencer. Sequence comparison and splicing used software, such as BWA 26 (Burrow-Wheeler Aligner 26), SAMtools27 (sequence alignment/map 27), and Picard^1^, and the reference gene group was GRCh37/hg19. The WES data was deposited to the repository of Sequence ReadArchive (SRA), and the number is PRJNA800000^2^.

### Pathogenic Variant Validation and Detection

Filtered pathogenic variants of WES were validated by Sanger sequencing. The PCR primers were designed by Primer 5 (Table 1), and the PCR products were detected by 1% agarose gel electrophoresis. Sanger sequencing was performed at Majorbio (Beijing, China).

**TABLE 1:** Primers of PCR.

Name	Sequence (5′–3′)
*NDP*-E1-F	TGGCATTCCCATTTGCTAGT
*NDP*-E1-R	AGGATGAAATGCTCGGTTTG
*NDP*-E2-F	CAGCCTTTGCTAATGACGCTCTA
*NDP*-E2-R	GCCCTCCAAGAAGTATGTTCCAC
*NDP*-E3-F3	TGGATGGGACAACTGTAGAGGCA
*NDP*-E3-R3	GCAGCAATGGCAACCTTAGACCA
F1	TTACTTCTCCCATGACCTGCTCT
R1	TGTTGTTAGTGTTCCGTGTCCCT
F2	CACAGCCAATGATGGGAGGGTAG
R2	GCAAAGTATGGGAGTGGGAGGAA
F3	AAGTTGGAAGAACCAGCAGAAGG
R3	GTGCGTTGAAACAAGCAAAGAGT
F4	CTGTGCTCTATGCCGTCTTCTCA
R4	CCTTCCACGTTCCTTCTCAAACT

## Results

### Clinical Manifestations of the Patients

**FIGURE 2:**
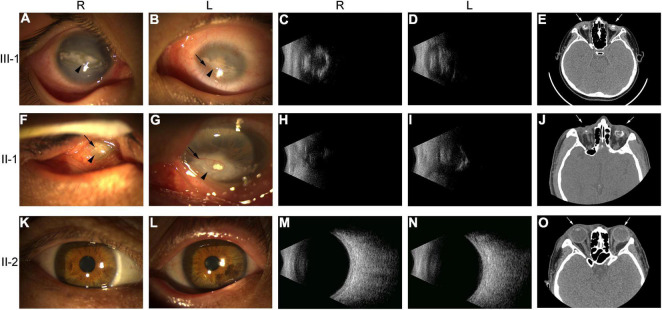
Ophthalmologic examination of members III-1, II-1, and II-2 of the ND family. **(A,B,F,G,K,L)** Anterior segment photography of III-1, II-1, and II-2. Black arrows indicated neovascularization; arrowheads indicated keratoleukoma. **(C,D,H,I,M,N)** Vitreous supersonic inspection. **(E,J,O)** CT inspections of the ears and eyes. White arrows indicated eyes. R, right eye; L, left eye.

The propositus (III-1) was a 10-year-old boy, and the pedigree was shown in Figure 1. III-1 had no light sensation in both eyes at birth, with progressive enophthalmos; the eyeball shrunk. The left cornea was small and opaque, and neovascularization grew into the peripheral cornea. The anterior chamber could not be observed due to the large range and deep involvement of corneal opacity. The corneal structure and morphology of the right eye were relatively normal, except that plaque opacity in the central corneal was distributed in horizontal strips. The transparency of the peripheral corneal was normal, but intraocular structure, such as iris, was still unclear (Figures 2A,B). Ultrasound B-scan examination revealed shrunk and small bilateral eyeballs, disordered intraocular structure, calcification and massive vitreous opacity, and unrecognizable retina (Figures 2C,D). CT examination showed irregular shape and reduced volume of bilateral eyeballs and patchy calcification (Figure 2E). The symptoms of the propositus and II-1 were very similar. No light sensation was experienced in both eyes of II-1, the eyeballs shrunk, the center of the cornea was porcelain-white opacity, and neovascularizations were found in the left eye that grew from the nasal side to the center and then surrounding the central calcificated area. Part of the iris was shown through the surrounding transparent cornea, and it was normal. Other intraocular structures could not be observed. Neovascularizations grew from the temporal side into the center of the cornea in the right eye, and other intraocular structures could not be observed (Figures 2F–J). The bilateral eyes of the propositus’s mother had normal size and structure, with transparent cornea, appropriate anterior chamber depth, clear aqueous humor, round pupil, transparent lens and vitreous body, and normal retina (Figures 2K–O).

**FIGURE 3:**
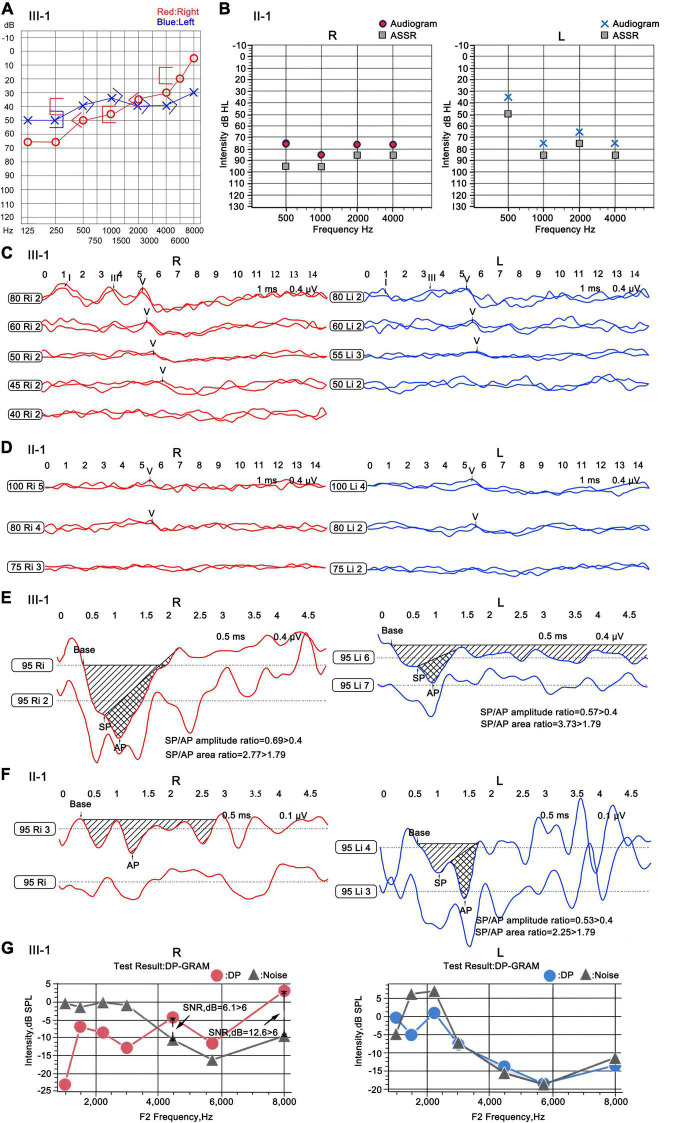
Audiology examination for the patients of the ND family. **(A)** Pure tone audiometry of III-1. “
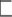
” and “
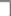
” indicated that contralateral ear was masked; “<” and “>” indicated bone conduction thresholds; “
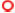
” and “
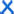
” indicated air conduction thresholds. **(B)** ASSR of II-1. Base, baseline; “
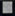
”: the thresholds of ASSR; “
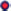
” and “
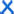
”: the calibrated response threshold of ASSR (Audiogram). **(C,D)** ABR of III-1 and II-1. **(E,F)** ECochG of III-1 and II-1. Base, baseline; SP, summating potential; AP, compound action potential. **(G)** DPOAE of III-1. DP, DPOAE amplitude; Noise, background noise amplitude; SNR, signal noise ratio; SNR > 6 dB indicates that the test is pass. R, right ear; L, left ear.

III-1 had normal ear structure, growth, and behavior and no peripheral vascular disease. Pure tone audiometry showed moderate sensorineural hearing loss in both ears, with evident low frequency hearing loss. The average hearing threshold of speech frequency (500; 1,000; and 2,000 Hz) was 55 dB, and the high-frequency (4,000∼ 8,000 Hz) hearing loss was mild; the hearing threshold map showed an upward pattern (Figure 3A). The wave I/III/V of ABR could be recorded at 80 dB threshold for both ears, and the thresholds of wave V were 55 dB for the left ear and 45 dB for the right ear (Figure 3C). The results of ECochG showed that the summating potential (SP)/compound action potential (AP) amplitude ratio was 0.69 for the right ear and 0.57 for the left ear (Normal value: 0.4); the SP/AP area ratios were 2.77 and 3.73 (Normal value: 1.79), which were abnormal (Figure 3E). Except at 4,444 and 8,000 Hz frequencies in the right ear, the DPOAE test failed at almost all frequencies in both ears (Figure 3G), suggesting the presence of lesions in the outer hair cells of the bilateral cochlea. However, the corresponding frequency positions of the right ear remained normal. Thin-slice CT scan showed that the structure of both ears was normal (the results were not shown).

**FIGURE 4:**
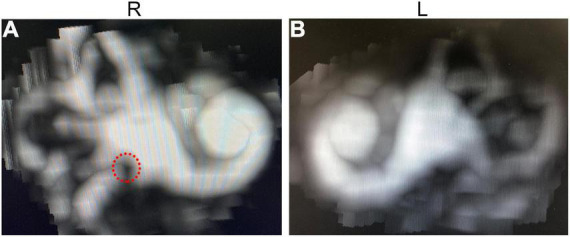
MIP images of MRI for III-1 following intravenous gadolinium (Gd) injection. **(A)** Image of the right ear. **(B)** Image of the left ear. The red dotted circle: showed distensible sacculus in the vestibular pool. R, right ear; L, left ear.

Patient II-1 was 39 years old. According to the family description, the patient had normal hearing when he was young, and hearing loss occurred when he was approximately 20 years old; the condition gradually worsened, and he had difficulty in communicating with words. He had normal ear structure, normal growth, and development, and not peripheral vascular disease. Due to the patient’s difficulty in communication, pure tone audiometry could not be performed, and air-conducting m-ASSR examination showed severe sensorineural hearing loss at almost all the frequencies in both ears. At 500 Hz, the response threshold of the right ear was 75 dB, and that of the left ear was only 35 dB. In the frequency region of 1,000–4,000 Hz, the response threshold of binaural exceeded 75 dB except for the 65 dB of the left ear at 2,000 Hz (Figure 3B). ABR examination indicated elevated threshold, only wave V was presented, and its threshold was 80 dB in both ears (Figure 3D). The results of ECochG showed that the SP/AP amplitude ratio was 0.53 and the SP/AP area ratio was 2.25 for the left ear; both ratios were abnormal, and the right ear cannot be presented (Figure 3F). The ECochG results of the two patients suggested they had endolymphatic hydrops (EH) with possibility. In view of both of the two patients showed the same abnormal symptoms, and after 1 year, reexamination of ECochG for III-1 showed the similar abnormal symptoms (data didn’t shown). Together, we determine the two patients had EH with high possibility. To conform this symptom, temporal bone MRI for III-1 was performed 4–6 h following an intravenous gadolinium (Gd) injection, then MR (magnetic resonance) maximum intensity projection (MIP) images were created. The images showed that there was a distensible sacculus in the vestibular pool of the right ear, while there had no this character in the left ear (Figure 4). These results indicated there had EH in the inner ear.

**FIGURE 5:**
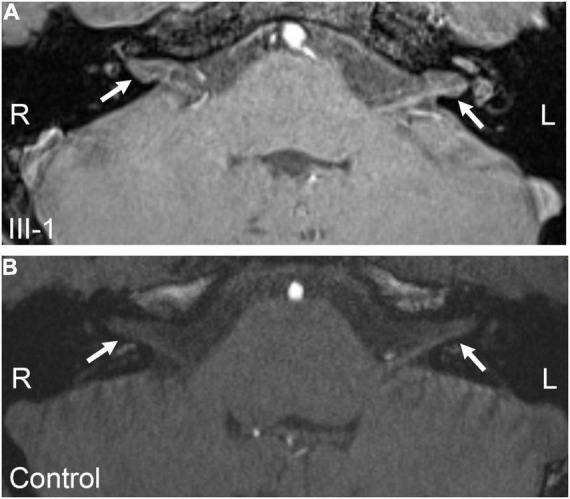
MRI of internal auditory canal. **(A)** Image of III-1. **(B)** Image of normal control. The arrows marked the auditory nerve. R, right; L, left.

The cognitive function of the patients was evaluated by MMSE and MOCA scales, and the spatial orientation, long-term memory, and computational ability decreased in III-1; the visual–spatial and logical ability tests could not be completed due to visual problems. II-1 could not be evaluated by scales due to difficulty in communication. The craniocerebral MRI results of II-1 showed few ischemic lesions in the brain, and the structure of hippocampus and fronto temporal cortex was normal without obvious atrophy (Supplementary Figure 1). However, a very recent study reported that an unexpected enhancement of several cranial nerves (CN): III; V, VII, VIII, IX, X, and XI were found in ND patients through MRI (Jokela et al., 2022). So the MRI of internal auditory canal for III-1 was performed, and the results showed that the acoustic nerves (VIII) were thick comparing to the normal control (Figure 5). In this family, I-1 was 68 years old and II-2 was 38 years old; their related symptoms were normal, and other members were normal.

### *NDP* Deletion Was Identified in the Norrie Disease Family

**FIGURE 6:**
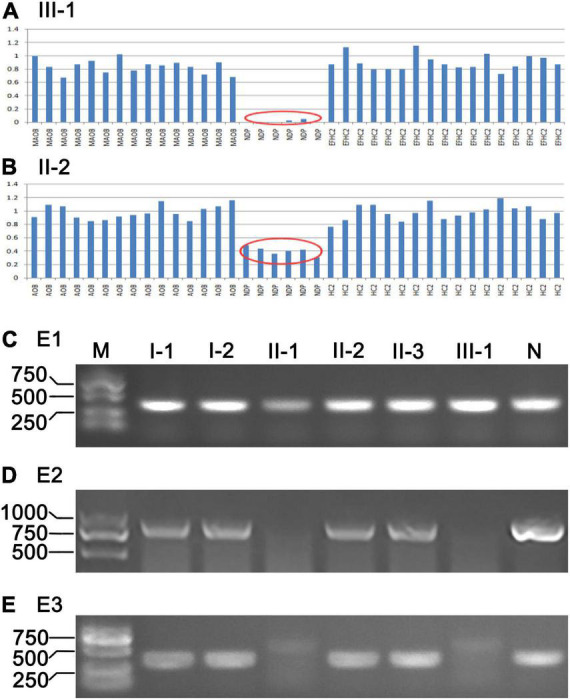
Results of WES and PCR. **(A,B)** Results of WES of III-1 and II-2, respectively. Red oval tag indicates exons of *NDP* were deleted. **(C–E)** PCR products of exon 1–3 (E1, E2, and E3) of *NDP* of the ND family; M, DNA ladder (DL2000); N, normal control.

WES was performed on three individuals of the pedigree, including II-1, II-2, and III-1 (Figure 1). The results indicated that II-1 and III-1 were hemizygous deletion of the *NDP* gene located on the X chromosome (Figure 6A, results of II-1 were not shown), and II-2 was heterozygous deletion (Figure 6B). We amplified exon1, exon2, and exon3 of *NDP* through PCR method to verify the results of WES. The results showed that the PCR products of E1 were present in all family members (Figure 6C), and no PCR products of E2 and E3 were found in II-1 and III-1; the target bands in other members of the family were all amplified (Figures 6D,E). These results indicated that E1 was not deleted in the two patients, E2 and E3 were deleted, but the hemizygous deletion in women and the exact location of the missing fragment needed to be identified.

### Location of *NDP* Deletion and Identification of Heterozygous Deletion

**FIGURE 7:**
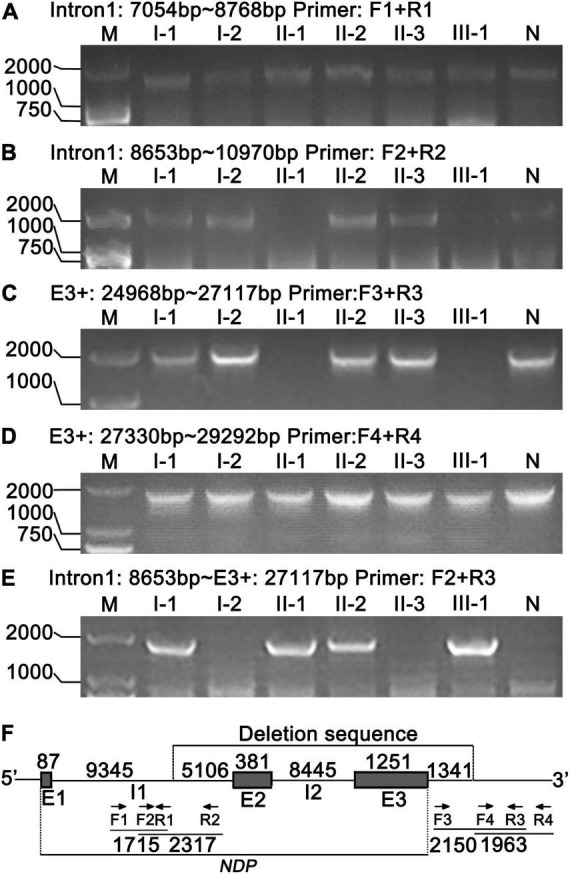
Location and identification of *NDP* deletion by PCR. **(A,B)** 5′ end location of *NDP* deletion. Primer pairs: F1, R1 and F2, R2, respectively. **(C,D)** 3′ end location of *NDP* deletion. Primer pairs: F3, R3 and F4, R4, respectively. **(E)** Identification of *NDP* deletion. Primer pairs: F2, R3. **(F)** Schematic of *NDP* deletion. E1, E2, and E3: exons 1–3 of *NDP*; I1, I2: introns 1–2 of *NDP*; arrows: primers; numbers: base count of each region.

We designed a series of PCR primers in the intron1 and downstream of E3 (Table 1 and Figure 7F) to accurately locate the large fragment deletion of *NDP*. The results showed no PCR products in the region 8,653–10,970 bp of *NDP* in II-1 and III-1 (Figure 7B), and the upstream of this region had target bands (7,054–8,768 bp) (Figure 7A), but the downstream of this region in the two patients had no target bands (results were not shown), indicating that the 5 ‘end boundary of *NDP* deletion was at 8,653–10,970 bp of *NDP*. Furthermore, the region 353–2,502 bp downstream the 3 ’end of *NDP* in II-1 and III-1had no amplified products, and the target products were present in the adjacent segment 2,715–4,677 bp, indicating that the downstream deletion site of *NDP* was within the region of 353–2,502 bp downstream of its 3′ end (Figures 7C,D).

Then, we selected the primers F2 and R3 to PCR, which might be in undeleted regions on both sides of the delete fragment. The results showed that approximately 1,800 bp fragments were amplified in I-1, II-1, II-2, and III-1, and no products were produced in other members (Figure 7E). Sequencing of this segment showed that the precise site of deletion was located 9,432 bp from *NDP*’s 5’end and extended to its downstream 1,341 bp of the 3’end of the gene, representing 16,525 bp, as shown in Figure 7F. At the same time, Figure 7E illustrates that the four members of I-1, II-1, II-2, and III-1 had chromosomes with *NDP* deletion. Among them, I-1 and II-2 also had a normal *NDP* gene (Figures 7B,C). Thus, they were heterozygous deletions also called carriers. II-1 and III-1 had no normal *NDP* gene; thus, they were patients (Figures 7B,C).

## Discussion

In addition to serious ophthalmologic defects, ND patients often show symptoms of progressive deafness. Some scholars characterized the audiologic phenotype of ND and found that ND patients generally showed mild sensorineural asymmetric and high-frequency hearing losses when they reach puberty (8 years old at the earliest, most 14 years old), and then developed into severe, symmetric, flat loss at approximately 35 years old. However, after severe hearing loss from approximately 35 to nearly 60 years old, the progress became stable (Halpin et al., 2005; Halpin and Sims, 2008). Similar to humans, the ND knockout mice have progressive hearing loss leading to profound deafness (Rehm et al., 2002). In this study, including typical ocular symptoms of ND disease, both patients showed obvious hearing loss. Hearing loss of the propositus was mild and asymmetrical between the left and right ears, which consistent with the general audiological characteristics of ND patients. However, the hearing loss of the propositus in the low frequency is more serious than that in the high frequency, which is different from the severe high-frequency hearing loss in ND patients in the previous reports.

Endolymph is closely related to vestibular and auditory sensors, and its production and absorption reflux is a circulatory process. If the reflux is blocked or absorption is impaired, then it can lead to excessive endolymphatic retention known as EH. EH can cause tinnitus, deafness, and vertigo in vestibular and auditory organs. Distension of the membranes in EH starts within the apex, and then the accumulated endolymph extends to the middle and base turn because the basilar membrane is wider and softer in the apex than that in the base of the cochlea (Yamashita and Schuknecht, 1982). Therefore, hearing loss caused by EH, such as Meniere’s disease, often starts from the low-frequency region and gradually develops to the high-frequency region (Gluth, 2020). In this study, ECochG examination suggested that EH was possible in the propositus and his uncle. Furthermore, gadolinium-enhanced MRI of ear for the propositus showed that there was a distensible sacculus in the vestibular pool of the right ear, which demonstrated there had EH in right inner ear of III-1. And for the ECochG examination of left ear also showed abnormal results, we speculate there might had slighter EH than that in the left ear, thus it wasn’t presented in the MRI imaging. As to the uncle, gadolinium-enhanced MRI wasn’t performed, but from the more serious ECochG results, we deduced that he also had EH in the inner ear. EH might be still in its early stage because of the young age of the propositus; thus, EH might mainly appeared at the apex of the cochlea and caused low-frequency damage initially. On the contrary, the uncle’s EH may have been developed for many years, it may have been extended from the apex to the base; and the audiologic phenotype of the two patients just be consistent with the EH process. The symptoms of EH have not been reported in ND patients so far. However, according to previous reports of ND patients that the development trend of hearing loss from high frequency to low frequency (Halpin et al., 2005), we can infer that these patients should not have EH even without the relevant examination. However, many other reports on hearing of ND patients had no EcochG examinations and did not describe auditory characteristics. These patients may conform to the hearing loss characteristics of general ND patients, that is without EH, but we cannot rule out the possibility of EH. There might had two possibilities about the correlation of EH and ND as follows: one, EH may only be the individual phenomenon of the present study, because specific genetic background contained some modifier gene in this family and *NDP* mutation alone does not cause EH. Another possibility is that EH is an uncommon phenotype of ND, which was missed due to insufficient attention in previous studies. We hope that future researchers can focus on this phenotype and further confirm this symptom. In summary, the symptom of EH in ND patients were reported for the first time; it may be one of the mechanisms of hearing loss of ND patients. These discoveries expanded the understanding of ND, and provided a new idea for the treatment of ND patients.

The mechanism of EH causing hearing loss has also been reported in many studies, and EH mediates the release of reactive oxygen species and nitric oxide to trigger apoptosis pathways via caspase-dependent and caspase-independent pathways, resulting in the reduction of helical ganglia (Merchant et al., 2005). In Phex^±^ mice, a model of EH, the loss of helical ganglion cells shows a topographical pattern (that is, starting at the apex of the cochlea and moving toward the base); this pattern mirrors the progression of EH. Interestingly, precedes the eventual loss of ganglion cells, the expression of apoptosis markers, such as caspase 3, 8, and 9, increased in spiral ganglion cells correspondingly (Semaan et al., 2013; Nakashima et al., 2016). In the present study, the audiological examination and the development history of hearing loss in two ND patients showed that the brainstem hearing area of this family was normal, and the problem was cochlear injury. In addition, most frequencies of DPOAE detection of the propositus failed to pass, indicating that hair cells were seriously damaged, probably due to EH.

Impairment of cochlear blood flow is observed in a wide variety of hearing disorders, including loud sound-induced hearing loss (endothelial injury) and aging-related hearing loss (lost vascular density). The study on ND knockout mice found that the stria vascularis was the most affected structure. With the increase of age, disorganization and enlarged vessels were observed in the stria vascularis, as was the lack of a well-developed capillary bed and eventual loss of two-thirds of the vessels in the stria vascularis. A gradual loss of outer hair cells was observed; it is particularly consistent with auditory threshold increases. As the hearing loss progressed, the spiral ganglion began to degenerate. These lesions were particularly pronounced in the apical portion of the cochlea (Rehm et al., 2002). So degeneration of vessels in the stria vascularis might be important pathogenesis of hearing loss of ND patients. Moreover, many findings demonstrated that circulatory disturbance may cause EH, which followed by striatal atrophy (Masutani et al., 1995). The same process may occur in the inner ear of Meniere patients, and the strial atrophy is also associated with extensive EH. Therefore, the impaired vascular stria microvascular system of Meniere patients is possible, leading to progressive degeneration of the inner ear over time (Yazawa et al., 1998). In the present study, the ND patients had EH and the corresponding hearing loss. Therefore, we speculated that the symptom of EH in the ND patients may also be related to the lesions of the inner ear microvascular system. However, the hearing loss in ND patients is more serious and happened earlier in the high frequency area, and no symptoms of EH in general. So, consistent with that EH phenotype might only presented in part of ND patients, EH might be the pathogenesis of hearing loss of part of ND patients.

**FIGURE 8:**
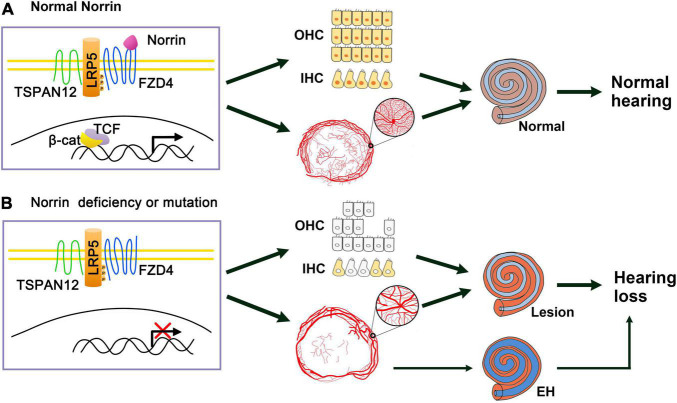
Schematic of Norrin deficiency causes deafness of ND patients through inactivating Wnt signal pathway. **(A)** When normal Norrin is existence, Norrin binds to the receptor complex FZD4/LRP5/TSPAN12 and Wnt signal pathway is activated, then transcription of the target genes turns on, thus maintaining the normal HCs and producing normal vascular system of the stria vascularum and hearing. **(B)** When Norrin is deficiency or mutation, which leads to destruction of Norrin/FZD4/LRP5/TSP12 complex and the failure of activation of Wnt pathway, which makes the key factors (marked with yellow and orange colors) of HCs be suppressed and HCs’ loss. At the same time, it induces the abnormal vascular system of the stria vascularum and then the lesions of the inner ear directly. And abnormal vascular system of the stria vascularum also can cause EH in some cases, then leads to EH related hearing loss. At last, all these three ways developed profound hearing loss.

Retinal vascular system defects often occur in ND and other related diseases caused by *NDP* mutation. Wnt signaling pathway plays a key role in the formation of blood vessels in various organs, including the eye. Various Wnt ligands are widely distributed in the retina and inner ear of developing mice (Yi et al., 2007; Iwai-Takekoshi et al., 2018). However, the role of Wnt signal in the retinal vascular system was not clarified until a study in 2004, which found that Norrin showed a high specific binding affinity to FZD4, the receptor of the Wnt pathway, and mutations of the two proteins caused similar vascular phenotypes. Moreover, this study reported that Norrin can induce the activation of FZD4 and LRP-dependent Wnt pathway, and revealed the role of Norrin-FZD4 signaling pathway in the development of ophthalmic and auricular vascular systems (Xu et al., 2004). Another study found that Norrin is a powerful trigger of FZD4 ubiquitination and induces the internalization of the Norrin receptor complex into the endolysosome chamber (Zhang et al., 2017). In addition, the Norrin/FZD4 signaling pathway requires another membrane protein, TSPAN12, which acts as an additional co-receptor to amplify the Wnt signal (Junge et al., 2009; Lai et al., 2017). In summary, the Norrin/FZD4/LRP5/TSPAN12 pathway has a unique and indispensable function in controlling retinal angiogenesis (Wang et al., 2019). In ND knockout mice, the vascular system of the cochlear stria vascularis was also abnormal. Thus, one of the main functions of Norrin in the ear is to regulate the interaction between the cochlea and blood vessels (Rehm et al., 2002). Norrin may regulate the blood vessels of the retina and cochlea through these pathways, which may be the molecular mechanism of the pathogenesis of ND. Therefore, we can speculate that Norrin’s mutation or deletion leads to the destruction of the Norrin/FZD4/LRP5/TSPAN12 pathway and the inactivation of Wnt pathway, causing abnormalities in the vascular system of the inner ear striated veins, which in turn makes the inner ear lesions. In addition, the abnormality of the stria vascularis can cause EH, which activates the apoptotic pathway, leading to the reduction of hair cells and spiral ganglia and causing EH-related hearing loss (Figure 8). However, in Hayashi’s recent report, Norrin deficiency made hair cells (HCs) loss the key transcription factors for HC maturation such as Pou4f3 and Gfi1 and a specialized myosin, Myo7a, required for normal function of HC stereocilia, which caused increasing HC loss and profound hearing loss. The molecular mechanism is that Norrin could orchestrates a transcriptional network for the maintenance and survival of HCs through activing Wnt signaling (Hayashi et al., 2021). So *NDP* could maintain the survival of HCs directly, which is another main molecular mechanism of ND (Figure 8).

Ye et al. (2011) detected the dynamic expression of *NDP* in the central nervous system and found that *NDP* was expressed in the ganglion eminence, hypothalamus, the subventricular zone, cerebellar primordium, and lateral olfactory tract of E15.5 mice. *NDP* was also expressed in the subventricular zone, cerebellum, amygdala, and lateral olfactory tract of P1 mice (Ye et al., 2011). McNeill et al. (2013) found that Norrin might play a role in regulating the proliferation of neural progenitor cells. These studies suggested that Norrin had important function in development and maintain of the central nervous system. In human, at least 50% of ND patients have developmental delay or progressive cognitive decline, often accompanied by psychotic features; some patients have epilepsy (Schuback et al., 1995; Smith et al., 2012; Okumura et al., 2015). Except for these symptoms, some unusual brain symptoms were also observed. For example, Warburg reported that an ND boy had chorea-like movements and without normal intelligence, and another patient from a different family makes numerous head movements while moving (Warburg, 1963). Mozo Cuadrado et al. (2020) reported that ND patients had nystagmus. A case reported cerebellar atrophy in an ND patient, and another study reported two ND patients from different sources, with brain and cerebellar atrophy (Liu et al., 2010; Rodriguez-Munoz et al., 2018). In this study, the propositus showed certain cognitive decline, and assessing his uncle was difficult due to communication difficulties caused by the severe hearing loss. However, even when he could be heard, he also could not respond, suggesting that the cognitive ability may have been severely damaged. The craniocerebral MRI of the uncle showed normal brain structure. However, a recent case report showed an unexpected enhancement of several cranial nerves (CN): III; V, VII, VIII, IX, X, and XI were found through MRI (Jokela et al., 2022). Second to this report, we also found the thick acoustic nerves (VIII) through the same examination. Although, we don’t know what effect of the enhancement of these nerves had on the ND patients or how they be formed, which would provide important clues to explore brain abnormalities of ND patients.

At present, no treatment is available for ND. Therefore, accurate genetic diagnosis and prenatal screening of this disease are particularly important. In this study, a large fragment deletion of *NDP* was found in an ND family by WES, and its deletion region was accurately located by PCR and Sanger sequencing. To diagnose the female carriers, especially the female fetuses or embryos in prenatal screening, WES or whole genome sequencing (WGS) is often used. However, when the deletion fragments have been identified, these methods are expensive and unnecessary. As for the reported Q-PCR methods (Sudha et al., 2018), we designed several primer pairs to detect this family and found that the results were very unstable and difficult to use. Therefore, in accordance with the characteristics of *NDP* with large segment deletion, we designed primers cleverly and used PCR methods to identify various genotypes, which were very stable and convenient. This PCR method provides a good idea for the diagnosis and prenatal screening of this ND family and other X-linked deletion diseases.

## Data Availability Statement

The datasets presented in this study can be found in online repositories. The names of the repository/repositories and accession number(s) can be found below: NCBI, SRA PRJNA800000.

## Ethics Statement

The studies involving human participants were reviewed and approved by the Ethics Committee of the Binzhou Medical University Hospital. Written informed consent to participate in this study was provided by the participants’ legal guardian/next of kin.

## Author Contributions

YG, ZL, and XZ performed the research and wrote the manuscript. JS and WL contributed to clinical examination. QX contributed to patient recruitment and data collection. SS and HZ analyzed the data. YW and QZ revised the manuscript. JC and XL contributed to project design and revised the manuscript. All authors contributed to the article and approved the submitted version.

## Conflict of Interest

The authors declare that the research was conducted in the absence of any commercial or financial relationships that could be construed as a potential conflict of interest.

## Publisher’s Note

All claims expressed in this article are solely those of the authors and do not necessarily represent those of their affiliated organizations, or those of the publisher, the editors and the reviewers. Any product that may be evaluated in this article, or claim that may be made by its manufacturer, is not guaranteed or endorsed by the publisher.

## Footnotes

1
http://broadinstitute.github.io/picard/

2
https://www.ncbi.nlm.nih.gov/sra/PRJNA800000

## References

[B1] Andarva M. Jamshidi J. Ghaedi H. Daftarian N. Emamalizadeh B. Alehabib E. (2018). A novel c.240_241insGG mutation in NDP gene in a family with Norrie disease. *Clin. Exp. Optom.* 101 255–259. 10.1111/cxo.12599 28922694

[B2] Chang T. H. Hsieh F. L. Zebisch M. Harlos K. Elegheert J. Jones E. Y. (2015). Structure and functional properties of Norrin mimic Wnt for signalling with Frizzled4, Lrp5/6, and proteoglycan. *Elife* 4:e06554. 10.7554/eLife.06554 26158506 PMC4497409

[B3] Chen J. Stahl A. Krah N. M. Seaward M. R. Dennison R. J. Sapieha P. (2011). Wnt signaling mediates pathological vascular growth in proliferative retinopathy. *Circulation* 124 1871–1881. 10.1161/CIRCULATIONAHA.111.040337 21969016 PMC3326389

[B4] Gluth M. B. (2020). On the Relationship Between Meniere’s Disease and Endolymphatic Hydrops. *Otol. Neurotol.* 41 242–249. 10.1097/MAO.0000000000002502 31746815

[B5] Halpin C. Owen G. Gutierrez-Espeleta G. A. Sims K. Rehm H. L. (2005). Audiologic features of Norrie disease. *Ann. Otol. Rhinol. Laryngol.* 114 533–538. 10.1177/000348940511400707 16134349

[B6] Halpin C. Sims K. (2008). Twenty years of audiology in a patient with Norrie disease. *Int. J. Pediatr. Otorhinolaryngol.* 72 1705–1710. 10.1016/j.ijporl.2008.08.007 18817988

[B7] Hayashi Y. Chiang H. Tian C. Indzhykulian A. A. Edge A. S. B. (2021). Norrie disease protein is essential for cochlear hair cell maturation. *Proc. Natl. Acad. Sci. U.S.A* 118:e2106369118. 10.1073/pnas.2106369118 34544869 PMC8488680

[B8] Iwai-Takekoshi L. Balasubramanian R. Sitko A. Khan R. Weinreb S. Robinson K. (2018). Activation of Wnt signaling reduces ipsilaterally projecting retinal ganglion cells in pigmented retina. *Development* 145:dev163212. 10.1242/dev.163212 30254141 PMC6240318

[B9] Jokela M. Karhu J. Nurminen J. Martikainen M. H. (2022). Multiple cranial nerve gadolinium enhancement in Norrie disease. *Ann. Neurol.* 91 158–159. 10.1002/ana.26274 34786741 PMC9299797

[B10] Junge H. J. Yang S. Burton J. B. Paes K. Shu X. French D. M. (2009). TSPAN12 regulates retinal vascular development by promoting Norrin- but not Wnt-induced FZD4/beta-catenin signaling. *Cell* 139 299–311. 10.1016/j.cell.2009.07.048 19837033

[B11] Lai M. B. Zhang C. Shi J. Johnson V. Khandan L. McVey J. (2017). TSPAN12 Is a Norrin co-receptor that amplifies frizzled4 ligand selectivity and signaling. *Cell Rep.* 19 2809–2822. 10.1016/j.celrep.2017.06.004 28658627 PMC5533581

[B12] Lev D. Weigl Y. Hasan M. Gak E. Davidovich M. Vinkler C. (2007). A novel missense mutation in the NDP gene in a child with Norrie disease and severe neurological involvement including infantile spasms. *Am. J. Med. Genet. A* 143A 921–924. 10.1002/ajmg.a.31531 17334993

[B13] Liu D. Hu Z. Peng Y. Yu C. Liu Y. Mo X. (2010). A novel nonsense mutation in the NDP gene in a Chinese family with Norrie disease. *Mol. Vis.* 16 2653–2658. 21179243 PMC3002970

[B14] Masutani H. Nakai Y. Kato A. (1995). Microvascular disorder of the stria vascularis in endolymphatic hydrops. *Acta. Otolaryngol. Suppl.* 519 74–77. 10.3109/00016489509121874 7610896

[B15] McNeill B. Mazerolle C. Bassett E. A. Mears A. J. Ringuette R. Lagali P. (2013). Hedgehog regulates Norrie disease protein to drive neural progenitor self-renewal. *Hum. Mol. Genet.* 22 1005–1016. 10.1093/hmg/dds505 23201751

[B16] Merchant S. N. Adams J. C. Nadol J. B. Jr. (2005). Pathophysiology of Meniere’s syndrome: are symptoms caused by endolymphatic hydrops? *Oto.l Neurotol.* 26 74–81. 10.1097/00129492-200501000-00013 15699723

[B17] Michaelides M. Luthert P. J. Cooling R. Firth H. Moore A. T. (2004). Norrie disease and peripheral venous insufficiency. *Br. J. Ophthalmol.* 88:1475. 10.1136/bjo.2004.042556 15489496 PMC1772398

[B18] Mozo Cuadrado M. Barrio L. Zubicoa Eneriz A. Antonia Ardanaz Aldave M. (2020). Ocular manifestations of Norrie disease. *J. Fr. Ophtalmol.* 43 439–441. 10.1016/j.jfo.2019.09.023 32381368

[B19] Nakashima T. Pyykko I. Arroll M. A. Casselbrant M. L. Foster C. A. Manzoor N. F. (2016). Meniere’s disease. *Nat. Rev. Dis. Primers* 2:16028. 10.1038/nrdp.2016.28 27170253

[B20] Okumura A. Arai E. Kitamura Y. Abe S. Ikeno M. Fujimaki T. (2015). Epilepsy phenotypes in siblings with Norrie disease. *Brain Dev.* 37 978–982. 10.1016/j.braindev.2015.04.004 25944760

[B21] Rehm H. L. Zhang D. S. Brown M. C. Burgess B. Halpin C. Berger W. (2002). Vascular defects and sensorineural deafness in a mouse model of Norrie disease. *J. Neurosci.* 22 4286–4292. 10.1523/JNEUROSCI.22-11-04286.2002 12040033 PMC6758776

[B22] Rodriguez-Munoz A. Garcia-Garcia G. Menor F. Millan J. M. Tomas-Vila M. Jaijo T. (2018). The importance of biochemical and genetic findings in the diagnosis of atypical Norrie disease. *Clin. Chem. Lab. Med.* 56 229–235. 10.1515/cclm-2017-0226 28742514

[B23] Schuback D. E. Chen Z. Y. Craig I. W. Breakefield X. O. Sims K. B. (1995). Mutations in the Norrie disease gene. *Hum. Mutat.* 5 285–292. 10.1002/humu.1380050403 7627181

[B24] Seitz R. Hackl S. Seibuchner T. Tamm E. R. Ohlmann A. (2010). Norrin mediates neuroprotective effects on retinal ganglion cells via activation of the Wnt/beta-catenin signaling pathway and the induction of neuroprotective growth factors in Muller cells. *J. Neurosci.* 30 5998–6010. 10.1523/JNEUROSCI.0730-10.2010 20427659 PMC6632606

[B25] Semaan M. T. Zheng Q. Y. Han F. Zheng Y. Yu H. Heaphy J. C. (2013). Characterization of neuronal cell death in the spiral ganglia of a mouse model of endolymphatic hydrops. *Otol. Neurotol.* 34 559–569. 10.1097/MAO.0b013e3182868312 23462289 PMC3628741

[B26] Sims K. B. Lebo R. V. Benson G. Shalish C. Schuback D. Chen Z. Y. (1992). The Norrie disease gene maps to a 150 kb region on chromosome Xp11.3. *Hum. Mol. Genet.* 1 83–89. 10.1093/hmg/1.2.83 1301161

[B27] Smith S. E. Mullen T. E. Graham D. Sims K. B. Rehm H. L. (2012). Norrie disease: extraocular clinical manifestations in 56 patients. *Am. J. Med. Genet. A* 158A 1909–1917. 10.1002/ajmg.a.35469 22786811

[B28] Sudha D. Ganapathy A. Mohan P. Mannan A. U. Krishna S. Neriyanuri S. (2018). Clinical and genetic analysis of Indian patients with NDP-related retinopathies. *Int. Ophthalmol.* 38 1251–1260. 10.1007/s10792-017-0589-0 28602015

[B29] Talebi F. Ghanbari Mardasi F. Mohammadi Asl J. Lashgari A. Farhadi F. (2018). Identification of a novel missense mutation in the norrie disease gene: the first molecular genetic analysis and prenatal diagnosis of Norrie disease in An Iranian Family. *Cell J.* 20 290–292. 10.22074/cellj.2018.5090 29633608 PMC5893302

[B30] Wang Z. Liu C. H. Huang S. Chen J. (2019). Wnt Signaling in vascular eye diseases. *Prog. Retin. Eye. Res.* 70 110–133. 10.1016/j.preteyeres.2018.11.008 30513356 PMC6545170

[B31] Warburg M. (1963). Norie’s disease (atrofia bulborum hereditaria). *Acta. Ophthalmol. (Copenh)* 41 134–146. 10.1111/j.1755-3768.1963.tb03533.x 13998843

[B32] Warburg M. (1975). Norrie’s disease–differential diagnosis and treatment. *Acta. Ophthalmol. (Copenh)* 53 217–236. 10.1111/j.1755-3768.1975.tb01156.x 808085

[B33] Xu Q. Wang Y. Dabdoub A. Smallwood P. M. Williams J. Woods C. (2004). Vascular development in the retina and inner ear: control by Norrin and Frizzled-4, a high-affinity ligand-receptor pair. *Cell* 116 883–895. 10.1016/s0092-8674(04)00216-8 15035989

[B34] Yamashita T. Schuknecht H. F. (1982). Apical endolymphatic hydrops. *Arch. Otolaryngol.* 108 463–466. 10.1001/archotol.1982.00790560001001 7103821

[B35] Yazawa Y. Kitano H. Suzuki M. Tanaka H. Kitajima K. (1998). Studies of cochlear blood flow in guinea pigs with endolymphatic hydrops. *ORL. J. Otorhinolaryngol. Relat. Spec.* 60 4–11. 10.1159/000027554 9519374

[B36] Ye X. Smallwood P. Nathans J. (2011). Expression of the Norrie disease gene (Ndp) in developing and adult mouse eye, ear, and brain. *Gene. Expr. Patterns* 11 151–155. 10.1016/j.gep.2010.10.007 21055480 PMC3061303

[B37] Yi H. Nakamura R. E. Mohamed O. Dufort D. Hackam A. S. (2007). Characterization of Wnt signaling during photoreceptor degeneration. *Invest. Ophthalmol. Vis. Sci.* 48 5733–5741. 10.1167/iovs.07-0097 18055826 PMC2330018

[B38] Zhang C. Lai M. B. Khandan L. Lee L. A. Chen Z. Junge H. J. (2017). Norrin-induced Frizzled4 endocytosis and endo-lysosomal trafficking control retinal angiogenesis and barrier function. *Nat. Commun.* 8:16050. 10.1038/ncomms16050 28675177 PMC5500887

