# Supplementary material for: Endolymphatic Hydrop Phenotype in Familial Norrie Disease Caused by Large Fragment Deletion of NDP
Source: Front Aging Neurosci. 2022 Apr 18;14:771328. doi: 10.3389/fnagi.2022.771328 (PMC9062296; doi:10.3389/fnagi.2022.771328)
Supplement: Supplementary file 1 [file Image_1.pdf]

Supplement Figures

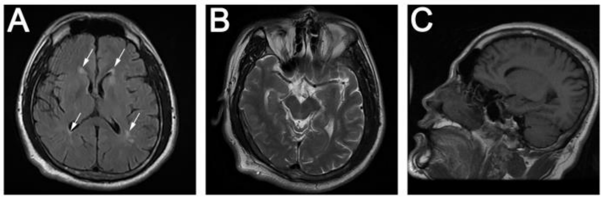

Supplementary Figure 1 | MRI of II-1
(A), (B): Transverse view of craniocerebral MRI. Arrows: ischemic lesions. (C) Sagittal l plane view of MRI.
